# Regulation of positive and negative emotion: effects of sociocultural context

**DOI:** 10.3389/fpsyg.2013.00259

**Published:** 2013-07-03

**Authors:** Sara A. Snyder, S. Megan Heller, Daniel S. Lumian, Kateri McRae

**Affiliations:** ^1^Department of Clinical and Counseling Psychology, Teachers College, Columbia UniversityNew York, NY, USA; ^2^Department of Psychology, University of DenverDenver, CO, USA; ^3^Department of Anthropology, University of California Los AngelesLos Angeles, CA, USA; ^4^Department of Psychology, University of California Los AngelesLos Angeles, CA, USA

**Keywords:** emotion regulation, cognitive reappraisal, expressive suppression, Burning Man, social context, cultural context, positive affect, negative affect

## Abstract

Previous research has demonstrated that the use of emotion regulation strategies can vary by sociocultural context. In a previous study, we reported changes in the use of two different emotion regulation strategies at an annual alternative cultural event, Burning Man (McRae et al., 2011). In this sociocultural context, as compared to typically at home, participants reported less use of expressive suppression (a strategy generally associated with maladaptive outcomes), and greater use of cognitive reappraisal (a strategy generally associated with adaptive outcomes). What remained unclear was whether these changes in self-reported emotion regulation strategy use were characterized by changes in the regulation of positive emotion, negative emotion, or both. We addressed this issue in the current study by asking Burning Man participants separate questions about positive and negative emotion. Using multiple datasets, we replicated our previous findings, and found that the decreased use of suppression is primarily driven by reports of decreased suppression of *positive* emotion at Burning Man. By contrast, the increased use of reappraisal is not characterized by differential reappraisal of positive and negative emotion at Burning Man. Moreover, we observed novel individual differences in the magnitude of these effects. The contextual changes in self-reported suppression that we observe are strongest for men and younger participants. For those who had previously attended Burning Man, we observed lower levels of self-reported suppression in both sociocultural contexts: Burning Man and typically at home. These findings have implications for understanding the ways in which certain sociocultural contexts may decrease suppression, and possibly minimize its associated maladaptive effects.

## Introduction

Functionalist approaches emphasize that emotions can promote quick, adaptive responses. However, sometimes our emotions are not appropriate for the environment that we are in, and consequently require active management. Emotion regulation refers to the various ways that individuals can manage, or control their emotional responses. The process by which we influence the type of emotions we have and how we express them is termed emotion regulation (Gross, 1998b; Gross et al., 2011). Emotion regulation helps us to match our environment and respond in more socially and contextually appropriate ways to enhance social acceptability and desirability (Szczurek et al., 2012). Successful use of emotion regulation is generally linked to adaptive functioning, and there are several strategies that individuals can deploy when attempting to use emotion regulation to change their emotion (Gross, 2002). Below, we review the literature on two strategies: expressive suppression and cognitive reappraisal. First, we will examine their short-term effects in experimental settings, as well as the long-term outcomes associated with their use. We then discuss the use of these strategies as they pertain to positive and negative emotion. Finally, we will outline the known effects of sociocultural context on strategy use.

### Expressive suppression

*Expressive suppression* is defined as the inhibition of emotion expression, such that an outside observer would be unaware of an individual's internal emotional experience (Gross, 1998a). Suppression can be used in interpersonal communication as a self-protective tool. By concealing negative expressions, for example, an individual can avoid unwanted questions or concern from other communicators on a daily basis (Butler et al., 2007). Unfortunately, suppression does not always lead to the desired changes in emotional experience. For example, experimental studies of suppression demonstrate that a negative emotional experience is only moderately diminished, or not changed at all, by the use of suppression (Stepper and Strack, 1993; Gross and Levenson, 1997; Egloff et al., 2006). Physiologically, suppression of negative emotion leads to paradoxical *increases* in central, peripheral, and sympathetic cardiovascular activation (Gross, 1998a; Goldin et al., 2008). Therefore, suppression is thought to lead to poor long-term health outcomes (Mauss and Gross, 2004; Nezlek and Kuppens, 2008). Those who use suppression frequently report lower levels of both positive affect and subjective well-being, along with greater levels of negative affect, and more depressive symptoms (Gross and John, 2003; Moore et al., 2008; Aldao et al., 2010). Long-term maladaptive effects of using suppression are also evident in individuals working in specific industries who are expected to display a certain countenance as part of their job responsibilities. Previous scholars have referred to the need for suppression as “face work,” referring to the act of expressing oneself in ways that work to maintain a positive social image and story of oneself (Goffman, 1955), and used the term “emotional labor” to describe the demands on individuals working in specific industries who are expected to express and suppress emotion as part of their job (Hochschild, 1983; Pierce, 1995). Considering this convergent evidence, expressive suppression is generally thought to be a relatively maladaptive emotion regulation strategy.

### Cognitive reappraisal

Unlike suppression, *cognitive reappraisal* uses active thinking to change emotion expression and experience (Gross, 1998b). Reappraisal refers to the reinterpretation or reframing of an emotional event in a way that changes the emotional meaning of the situation, and therefore can change emotions by changing how an individual is thinking (Lazarus, 1991; Gross and Levenson, 1997). Experimentally, reappraisal has been used to both increase and decrease an individual's subjective experience of both positive and negative emotion (Gross, 1998a; Ochsner and Gross, 2004; Kim and Hamann, 2007; Giuliani et al., 2008). Reappraisal can also be used to impact both peripheral and central measures of physiological responding in accordance with the desired goal of regulation (Jackson et al., 2000; Hajcak et al., 2006; Ray et al., 2010; Kim and Hamann, 2012). Frequent use of reappraisal has also been associated with more adaptive outcomes, including greater levels of positive affect and well-being, lower levels of negative affect, and fewer depressive symptoms (Gross and John, 2003; Aldao et al., 2010). Therefore, reappraisal is thought to be a relatively successful and adaptive regulation strategy.

### Emotion regulation of positive and negative emotions

There is a fair amount of convergent evidence about the experimental effects and long-term consequences of suppression and reappraisal. However, there is reason to believe that the outcomes of these regulation strategies, particularly suppression, may differ when used to change positive and negative emotion. Suppression appears to operate somewhat differently on positive and negative emotion. Inhibiting the expression of positive emotion results in decreased subjective experience of positive emotion, whereas inhibiting the expression of negative emotion does not have this effect and, paradoxically, results in additional increases in some measures of negative emotion (Gross, 1998a; Butler et al., 2003; Goldin et al., 2008). Therefore, the use of suppression on positive and negative emotion may have undesirable consequences, but operate through different mechanisms (decreased positive emotion experience vs. undiminished negative emotion experience).

Taking a look at reappraisal, recent work has begun to distinguish between using reappraisal to change the experience of negative emotion or positive emotion (Shiota and Levenson, 2009, 2012; McRae et al., 2012). There are different experiential and physiological effects of using reappraisal to decrease negative emotion compared with increasing positive emotion (McRae et al., 2012). In addition, there is some evidence that the ability to use reappraisal to increase positive emotion is more closely linked with adaptive outcomes than using it to decrease negative emotion (Troy et al., 2010). Despite these potentially important differences, few studies have examined the use of suppression and reappraisal to regulate positive and negative emotion separately.

### Sociocultural context and emotion regulation

Because the use of suppression and reappraisal is generally associated with maladaptive and adaptive outcomes, respectively, it is important to identify the situations in which individuals use suppression and reappraisal less and more frequently. One important contributor to the use of these strategies may be an individual's sociocultural context. Research on cultural differences in emotion regulation has demonstrated that individuals in Eastern cultures tend to use suppression more frequently than those in Western cultures (Matsumoto et al., 2008). Interestingly, this relatively increased use of suppression does not appear to be associated with maladaptive outcomes in Eastern cultures (Butler et al., 2007; Soto et al., 2011). One potential mechanism for this cultural difference may be the relative stability of social hierarchies in these different cultures. Individuals who find themselves in relatively stable, long-term oriented hierarchies are more likely to adaptively utilize suppression to maintain their position in the social order—more so than individuals operating in a context where an individual must advance more quickly than others in order to maintain his or her social status (Matsumoto et al., 2008). Less work has been done on cultural differences that are associated with changes in reappraisal use.

While broad characteristics of cultural variation in emotion regulation patterns may be somewhat informative, it is also important to examine how swift or dramatic *changes* in sociocultural context may demand that a person alters, or is flexible with, his or her emotion regulation strategies (Bonanno et al., 2004; Westphal et al., 2010). One study examined how students in the United States (US) regulate emotion during a stressful social transition, from high school to college. This study found that students reported using suppression more frequently during their first term of college than during their last term of high school, likely due to the destabilizing transition from familiar to unfamiliar. In addition, self-reported suppression use was a predictor for adverse social outcomes during the transition (Srivastava et al., 2009). No differences in self-reported reappraisal were observed during the transition to college.

Another study examined changes in emotion regulation brought about by a dramatic, temporary change in sociocultural context for individuals attending the Burning Man event (McRae et al., 2011). Burning Man is an annual art festival and alternative cultural gathering held for 1 week every summer in a Nevada desert. The organizers of the event actively encourage “radical self expression” from the 50,000 plus participants (Burning Man Organization, 2011). Some choose to wear elaborate costumes or colorful body paint, and often nothing at all. Hardworking participants often express themselves and contribute to the creative culture by producing elaborate art, sculptures, and shade structure, music, and dance (Chen, 2009, 2012a,b). This art is often interactive, and some is burned in massive fires at the end of the week as a form of group catharsis, including the iconic Burning Man figure himself. The event constitutes an alternative sociocultural context in that it is de-commercialized, operating without corporate sponsorship and running on a gift economy; meaning that goods and services are neither sold nor bartered, but shared freely among participants without expectations of a return gift (Kozinets, 2002; Kozinets and Sherry, 2005). Over the past quarter century, Burning Man's alternative cultural setting has been a site for social experimentation. Many participants view Burning Man as a social movement, not just a vacation destination, and they endeavor to export the values and traditions of Burning Man into their workplaces and local communities (Turner, 2009; Chen, 2011). The movement explicitly promotes a more creative culture of experimentation, not just in the artistic sense, but also by encouraging people to reinvent themselves and reimagine what it is to be an active participant in a social community.

Comparing these two contextual changes in terms of emotion regulation, it is important to note the ways that a transition from typical home life to Burning Man is unlike that from high school to college. Simply stated, both transitions entail removing oneself from the larger society for a time. The primary differences are that college is usually a setting for acquiring stable characteristics that will improve one's opportunities in an extant hierarchical society over an extended duration (typically 4 years), while Burning Man is a short-term setting (the event lasts 1 week) that fosters individual creativity, cultural experimentation, and collective reconstructing of society in alternative forms.

In the previous study, we identified Burning Man as a sociocultural context in which emotion regulation becomes more adaptive (McRae et al., 2011). We found that self-reported suppression was decreased at Burning Man compared to home, while self-reported reappraisal was increased at Burning Man compared to home. However, our previous study did not address whether these differences in emotion regulation strategy use were characterized by changes in the regulation of positive emotion, the regulation of negative emotion, or both. In addition, we did not previously test for differences in emotion regulation by age, gender, or previous experience at Burning Man (McRae et al., 2011).

### Present study

The primary purpose of the current study was to test for possible valence asymmetries underlying the changes in self-reported emotion regulation observed in the sociocultural contexts of Burning Man and typically at home, as well as examine individual differences in these changes. In the present study, we wanted to know whether the decreased use of suppression at Burning Man is characterized by decreased suppression of positive emotion, negative emotion, or both. In addition, we wanted to know whether the increased use of reappraisal at Burning Man is characterized by increased reappraisal of positive emotion, negative emotion, or both. We predicted decreased suppression and increased reappraisal at Burning Man, both with differential effects for the regulation of positive and negative emotion. Because the suppression of negative emotion is more prevalent than the suppression of positive emotion in everyday life (Gross and John, 2003), we predicted stronger decreases in suppression of negative emotion compared to the decreases in suppression of positive emotion at Burning Man. For reappraisal, based on the limited literature separating the reappraisal of positive and negative emotion, we had the prediction that participants at Burning Man would use reappraisal with the goal of creating or maintaining a high arousal emotional state (McRae et al., 2012), and therefore predicted greater increases in the reappraisal of positive emotion compared with increases in the reappraisal of negative emotion. Finally, we wanted to examine whether these context and valence interactions are comparable for individuals with differences in gender, age, and experience at Burning Man, because previous research has found differences in the use of suppression and reappraisal by age and gender (Gross and John, 2003).

## Method

### Participants

Participants for Studies 1–4 were recruited at the annual Burning Man event during four consecutive years: Study 1 (August 25th–September 1st, 2008; population 49,599), Study 2 (August 31st–September 7th, 2009; population 43,558), Study 3 (August 30th–September 6th, 2010; population 51,525), and Study 4 (August 29th–September 5th, 2011; population capped at 50,000) (Burning Man Organization, 2011). Institutional review boards at The University of California, Los Angeles (Studies 1–4), Stanford University (Studies 1–3), and The University of Denver (Studies 3 and 4) approved the collection and analysis of data for these four studies. This study is part of a collaboration among several researchers from the US and Canada who all work on an annual survey that is managed by the Burning Man organization and offered during the event. Each year's survey is different, but questions usually focus on basic demographic characteristics, participation in Burning Man, as well as our questions on emotion regulation. Participants were included if they provided answers for all of the items listed below. In addition, for Studies 2–4, participants were only included if they responded correctly to an item designed to ensure conscientious responding. This item read: “If you are reading this form carefully, please leave the response options below blank, but draw a circle around the first instance of the word “carefully” in this sentence.” Only participants who correctly omitted the response and circled the correct word were included. The final samples were comprised of 3472 participants for Study 1 (45.3% women, age not available), 2459 participants for Study 2 (45% women, mean age = 36.72, *SD* = 12.04), 3990 participants for Study 3 (46.5% women, mean age = 37.07, *SD* = 11.40), and 6306 participants for Study 4 (47.4% women, mean age = 35.17, *SD* = 11.50).

### Measures

To measure emotion regulation use, we used modified core items from the Emotion Regulation Questionnaire (ERQ; Gross and John, 2003). For Study 1, we used one item from the suppression scale (“I can control my emotions by not expressing them”) and another from the reappraisal scale (“I can control my emotions by changing the way I think about the situation”). Study 2 consisted of three emotion regulation questions, two suppression (“When I want to feel less negative emotion, such as sadness or anger, I make sure not to express them” and “When I am feeling positive emotions, such as joy or amusement, I am careful not to express them”) and one reappraisal (“I control my emotions by changing the way I think about the situation”). For Study 3, we asked about the use of regulation strategies for positive and negative emotion separately: suppression of positive and negative emotion (“When I am feeling positive emotions, I am careful not to express them” and “When I am feeling negative emotions, I make sure not to express them,” respectively) as well as reappraisal of positive and negative emotion (“When I want to feel more positive emotion (such as joy or amusement) I change what I am thinking about” and “When I want to feel less negative emotion (such as sadness or anger) I change what I am thinking about,” respectively). For Study 4 we asked about the use of regulation strategies for positive and negative emotion separately and with slightly different wording from Study 3. We asked about suppression of positive (“When I am feeling positive emotions, I am careful not to express them.”) and negative emotion (“When I am feeling negative emotions, I am careful not to express them”) as well as reappraisal of positive (“When I want to feel more positive emotion, I change the way I'm thinking about the situation”) and negative emotion (“When I want to feel less negative emotion, I change the way I'm thinking about the situation”). We have previously reported strong item-scale correlations using variations on these items before (McRae et al., 2011), and we were confident that, given time and space limitations, these single-item measures would be effective (like other single-item measures; see Robins et al., 2001; Gosling et al., 2003).

To assess the degree to which participants used each emotion regulation strategy in the four studies we used a 9-point Likert scale. The lowest score, a 1, was labeled “Not at all like me” and the highest score, a 9, was labeled “Very much like me” with a 5 labeled “Neutral.” Participants were instructed to write in the appropriate response in the two provided columns labeled “Off Playa” and “On Playa.” (“The Playa” is a common term referring to Black Rock City or Burning Man.) In multiple previous studies using the full ERQ (Gross and John, 2003; John and Gross, 2004) suppression and reappraisal were essentially unrelated, with correlations close to 0 and not significantly exceeding 0.11. We replicated this effect in Studies 1–4 at Burning Man (*r* = 0.04, *r* = 0.06, *r* = 0.002, *r* = 0.06; respectively) and for typical use at home (*r* = 0.03, *r* = 0.04, *r* = 0.04, *r* = 0.07; respectively).

### Procedure

Participants were individuals who attended the Burning Man event during 2008, 2009, 2010, or 2011 and completed the survey voluntarily. Blank survey forms were left in centralized, well-trafficked locations, and instructions on the top of the page invited participants to fill them out voluntarily. Participants returned the completed survey to marked receptacles in the same locations. In addition to the emotion regulation questions, the survey also included demographic questions (age, gender, place of residence, income, etc.) included for use by the event organizers. After the event, responses from the paper forms were entered into either a spreadsheet (Studies 1–3) or a data entry website (Study 4) by a team of researchers.

### Analysis

Values on individual items were transformed to percent of maximum possible (POMP) scores, which range from 0 to 100 to facilitate comparison with previous results (Cohen et al., 1999). POMP scores are always expressed as a percentage of the highest response option, and therefore facilitate the comparison of survey data when the scale is not consistent. For Studies 1–4, POMP scores for suppression and reappraisal were entered into a repeated measures general linear model (GLM) in SPSS with regulation strategy (suppression vs. reappraisal) and context (home vs. Burning Man) as repeated measures. For Studies 3 and 4, GLMs also included valence (positive vs. negative) as a repeated measure. Follow-up analyses investigated the effect of context separately for each valence and regulation strategy. For secondary analyses, we conducted separate analyses considering gender (men or women) as a between-subjects factor, age as a continuous covariate, and previous Burning Man experience (those who were there for the first year or those who had previously attended) as a between-subjects factor.

## Results

### Differences in emotion regulation by context

First, we examined differences in self-reported strategy use in the two contexts for Studies 1–4. We observed a replication of our previous findings (McRae et al., 2011)—a significant interaction between self-reported strategy use and context for Study 1, *F*_(1, 3471)_ = 354.26, *p* < 0.001 Cohen's *d* = 0.67; Study 2, *F*_(1, 2458)_ = 38.21, *p* < 0.001, Cohen's *d* = 0.25; Study 3 *F*_(1, 3989)_ = 154.79, *p* < 0.001, Cohen's *d* = 0.40; and Study 4, *F*_(1, 6305)_ = 846.90, *p* < 0.001, Cohen's *d* = 0.78. Follow-up tests indicated that these interactions were characterized by individuals reporting using suppression less frequently at Burning Man than typically at home for Study 1, *t*_(3471)_ = 19.28 *p* < 0.001, Cohen's *d* = 0.33; Study 2 *F*_(1, 2458)_ = 126.73, *p* < 0.001, Cohen's *d* = 0.47; Study 3, *F*_(1, 3989)_ = 150.55, *p* < 0.001, Cohen's *d* = 0.40; and Study 4, *F*_(1, 6305)_ = 311.09, *p* < 0.001, Cohen's *d* = 0.46.

**Figure 1:**
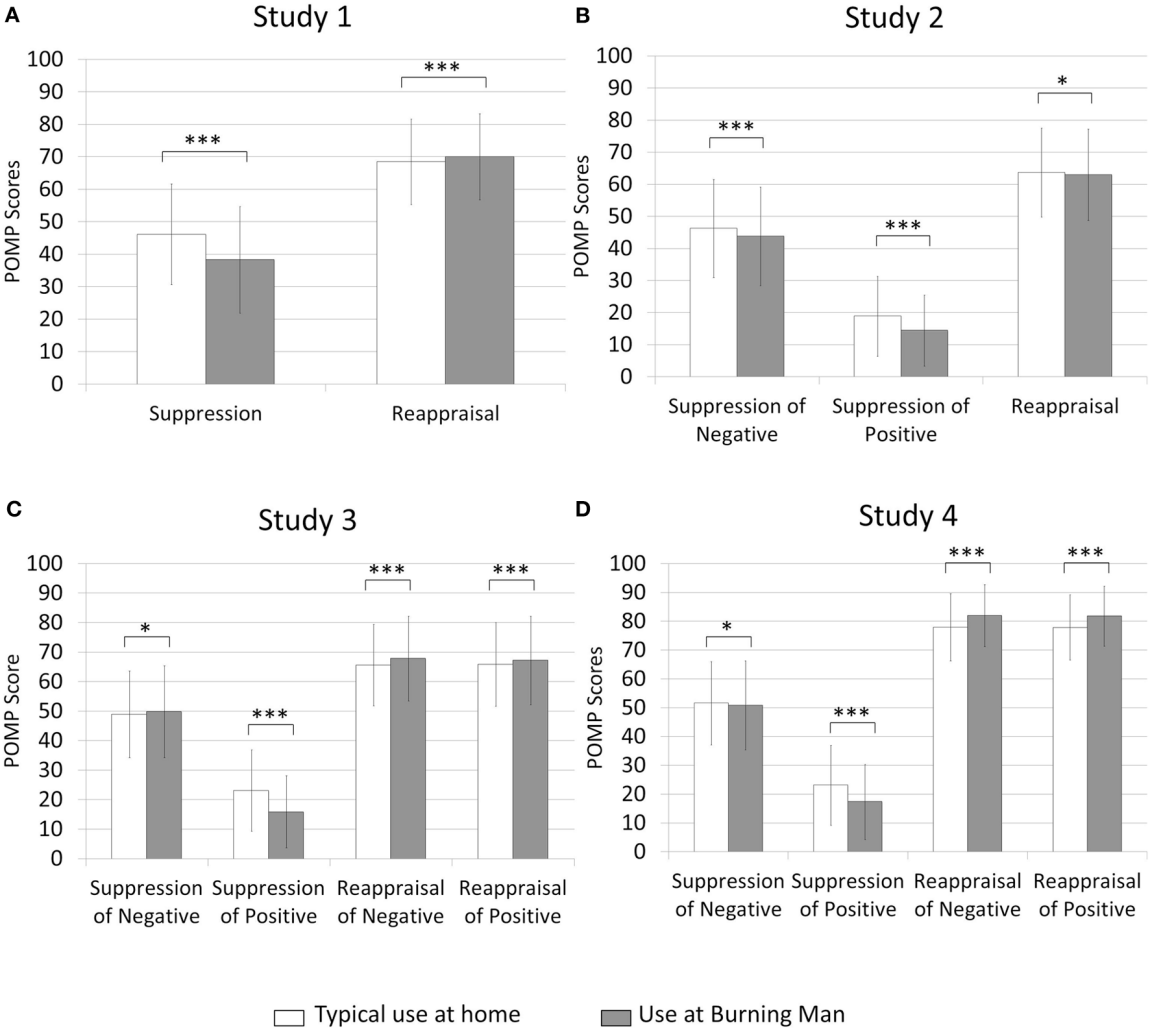
**Percentage maximum possible (POMP) scores indicating frequency of self-reported suppression and reappraisal use typically at home and at Burning Man in Studies 1–4 (panels **A–D**, respectively)**. For Studies 2–4 (panels **B–D**), suppression of positive and negative emotion was measured separately. For Studies 3–4 (panels **C–D**), reappraisal of positive and negative emotion was measured separately. Error bars indicate 1/2 the standard deviation from the mean in each direction. ^*^p < 0.05; ^***^p < 0.001.

By contrast, self-reported reappraisal use was greater at Burning Man compared with typically at home in Study 1, *t*_(3471)_ = 5.29, *p* < 0.001, Cohen's *d* = 0.09; Study 3, *F*_(1, 3989)_ = 31.61, *p* < 0.001, Cohen's *d* = 0.18; and Study 4, *F*_(1, 6305)_ = 618.45, *p* < 0.001, Cohen's *d* = 0.66. This is the same pattern we observed previously. In Study 2, we observed a relatively weak reversal of this effect indicating that participants reported using reappraisal less at Burning Man than typically at home, *t*_(2458)_ = 2.09, *p* < 0.04, Cohen's *d* = 0.04. For means, see Table 1 and Figure 1.

**Table 1:** **Group means for the primary analyses**.

**Strategy**	**Context**	**Valence**	**Study 1**	**Study 2**	**Study 3**	**Study 4**
Suppression	Burning Man	Positive	38.29 (30.99)	14.38 (22.23)	15.88 (24.52)	16.65 (25.26)
		Negative		43.73 (30.74)	49.81 (31.07)	50.85 (30.68)
	Typical Use at Home	Positive	46.03 (32.80)	18.86 (24.88)	23.08 (27.55)	22.62 (27.44)
		Negative		46.23 (30.57)	49.04 (29.33)	51.58 (28.89)
Reappraisal	Burning Man	Positive	70.01 (26.39)	62.95 (28.43)	67.15 (30.13)	81.60 (20.80)
		Negative		43.73 (30.74)	67.73 (28.91)	82.02 (21.18)
	Typical Use at Home	Positive	68.43 (26.50)	63.69 (27.70)	65.81 (28.44)	77.57 (22.72)
		Negative		46.23 (30.57)	65.43 (27.63)	77.84 (23.10)

Means for each POMP score is presented for each strategy, context (at Burning Man or typical use at home), and valence. For the years in which questions did not specify emotional valence, only a single mean is shown. Standard deviations appear in parenthesis.

### Regulation of positive and negative emotion

Next, we investigated the role of valence in the previously reported interaction between self-reported strategy and context. Because changes in suppression at Burning Man are more prominent than changes in reappraisal, we began by examining the suppression of positive and negative emotion separately at home and at Burning Man in Study 2. We observed an interaction between context and valence for suppression, *F*_(1, 2458)_ = 12.21, *p* < 0.001, Cohen's *d* = 0.14. Self-reported suppression decreased, both for positive, *t*_(2458)_ = 11.76, *p* < 0.001, Cohen's *d* = 0.24; and negative emotion, *t*_(2458)_ = 5.50, *p* < 0.001, Cohen's *d* = 0.11, at Burning Man compared to typically at home, but the magnitude of this decrease (as a difference score) was greater for the suppression of positive emotion compared with negative emotion, *t*_(2458)_ = 3.49, *p* < 0.001, Cohen's *d* = 0.07.

To examine whether the differential regulation of positive and negative emotion was also evident for reappraisal, we asked about the use of suppression and reappraisal to change positive and negative emotion separately for Studies 3 and 4. We observed a three-way interaction between self-reported strategy, context and valence for Study 3, *F*_(1, 3989)_ = 137.55, *p* < 0.001, Cohen's *d* = 0.38; and Study 4, *F*_(1, 6305)_ = 166.27 *p* < 0.001, Cohen's *d* = 0.34. This three-way interaction was characterized by an interaction between valence and context for suppression, for Study 3 *F*_(1, 3989)_ = 273.94, *p* < 0.001, Cohen's *d* = 0.54; and Study 4, *F*_(1, 6305)_ = 223.26, *p* < 0.001, Cohen's *d* = 0.38. Follow up analyses of this interaction between context and valence for suppression were consistent with the pattern observed in Study 2, indicating strong decreases in self-reported suppression of positive emotion at Burning Man compared to home in Study 3, *t*_(3989)_ = 22.54, *p* < 0.001, Cohen's *d* = 0.36; and Study 4, *t*_(6305)_ = 27.56, *p* < 0.001, Cohen's = 0.35. By contrast, contextual changes in self-reported suppression of negative emotion were not as strong in Study 4, *t*_(6305)_ = 2.48, *p* < 0.02, Cohen's *d* = 0.03, and even showed a weak reversal in Study 3, *t*_(3989)_ = 1.98, *p* < 0.05, Cohen's *d* = 0.03.

For reappraisal, we observed an interaction between context and valence in Study 3, *F*_(1, 3989)_ = 7.39, *p* < 0.008, Cohen's *d* = 0.09, but this was still a substantially smaller effect than the comparable interaction for suppression. Follow-up tests showed that participants reported increased reappraisal in order to *both* increase positive emotion, *t*_(3989)_ = 3.65, *p* < 0.001, Cohen's *d* = 0.06, and decrease negative emotion, *t*_(3989)_ = 6.27, *p* < 0.001, Cohen's *d* = 0.10, at Burning Man compared with typically at home. The difference in effect size for the self-reported reappraisal of positive and negative emotion (difference in Cohen's *d* = 0.04) was markedly smaller than any of the differences in the self-reported suppression of positive and negative emotion (smallest difference in Cohen's *d* = 0.13). We did *not* observe an interaction between context and valence for reappraisal in Study 4, *F*_(1, 6305)_ = 0.75, *p* = 0.39, Cohen's *d* = 0.02. Consistent with this, individuals reported using reappraisal more at Burning Man than typically at home, for both increasing positive emotion, *t*_(6305)_ = 21.26, *p* < 0.001, Cohen's *d* = 0.27, and decreasing negative emotion, *t*_(6305)_ = 22.36, *p* < 0.001, Cohen's *d* = 0.28, to similar extents. See Table 1 and Figure 1.

### Secondary analyses

To examine whether the interactions we report between context, self-reported regulation strategy, and valence were moderated by demographic and group variables, we examined separate models that tested for interactions with gender, age, and previous experience at Burning Man.

#### Gender

Consistent with previous results, we consistently observed an interaction between self-reported strategy use and gender in Study 1, *F*_(1, 3472)_ = 86.66, *p* < 0.001, Cohen's *d* = 0.32; Study 2, *F*_(1, 2457)_ = 59.90, *p* < 0.001, Cohen's *d* = 0.32; Study 3, *F*_(1, 3988)_ = 186.32, *p* < 0.001, Cohen's *d* = 0.44; and Study 4, *F*_(1, 6304)_ = 193.05, *p* < 0.001, Cohen's *d* = 0.36. This interaction was characterized by greater use of suppression in men than women in Study 1, *t*_(3470)_ = 10.63, *p* < 0.001, Cohen's *d* = 0.36; Study 2, *t*_(2457)_ = 8.85, *p* < 0.001, Cohen's *d* = 0.36; Study 3, *t*_(3988)_ = 10.09, *p* < 0.001, Cohen's *d* = 0.32; and Study 4, *t*_(6304)_ = 11.00, *p* < 0.001, Cohen's *d* = 0.28, and greater use of reappraisal in women than men in Study 2, *t*_(2457)_ = 2.88, *p* < 0.05, Cohen's *d* = 0.12; Study 3, *t*_(3988)_ = 9.45, *p* < 0.001, Cohen's *d* = 0.30; and Study 4, *t*_(6304)_ = 9.25, *p* < 0.001, Cohen's *d* = 0.23.

In Studies 1 and 2, this was the only significant effect of gender^1^. In Studies 3 and 4, we observed several two- and three- way interactions with gender, all of which were qualified by a four-way interaction between self-reported regulation strategy, context, valence and gender as a trend for Study 3, *F*_(1, 3988)_ = 3.75, *p* = 0.05, Cohen's *d* = 0.06; and Study 4, *F*_(1, 6304)_ = 4.94, *p* < 0.03, Cohen's *d* = 05. In both studies, this is best characterized as a three-way interaction between context, valence and gender for suppression, as a trend in Study 3, *F*_(1, 3988)_ = 3.43, *p* = 0.06, Cohen's *d* = 0.06; and Study 4, *F*_(1, 6304)_ = 10.79, *p* = 0.001, Cohen's *d* = 0.08. More specifically, this three-way interaction for suppression was characterized by the largest contextual change in suppression in men while suppressing positive emotion. In other words, the interaction between context and valence (greater contextual decreases in the self-reported suppression of positive than negative emotion) is true of both men in Study 3, *F*_(2134)_ = 163.77, *p* < 0.001, Cohen's *d* = 0.57; and Study 4, *F*_(3318)_ = 164.99, *p* < 0.001, Cohen's *d* = 0.46, and women in Study 3, *F*_(1854)_ = 110.39, *p* < 0.001, Cohen's *d* = 0.50; and Study 4, *F*_(2986)_ = 65.57, *p* < 0.001, Cohen's *d* = 0.30, but appears stronger in men than women. By contrast, we did not observe such a three-way interaction for reappraisal, all *p*s > 0.13. Means split by gender are in Table 2.

**Table 2:** **Impact of gender on emotion regulation usage at Burning Man and at home**.

**Strategy**	**Context**	**Gender**	**Valence**	**Study 1**	**Study 2**	**Study 3**	**Study 4**
Suppression	Burning Man	Women	Positive	32.92 (29.82)	10.74 (20.24)	12.46 (23.09)	12.58 (23.45)
			Negative		39.94 (30.91)	47.24 (31.70)	49.81 (30.56)
		Men	Positive	42.74 (31.24)	17.35 (23.33)	18.86 (25.34)	20.31 (26.25)
			Negative		46.82 (30.26)	52.04 (30.34)	51.78 (30.76)
	Typical Use at Home	Women	Positive	39.83 (32.77)	14.43 (23.00)	18.34 (26.26)	17.19 (25.32)
			Negative		42.69 (31.33)	46.12 (29.59)	50.39 (28.68)
		Men	Positive	51.16 (31.93)	22.48 (25.77)	27.20 (27.98)	27.50 (28.33)
			Negative		49.13 (29.63)	51.58 (28.87)	52.64 (29.04)
Reappraisal	Burning Man	Women	Positive	70.83 (25.92)	65.01 (28.10)	71.48 (28.39)	83.97 (18.79)
			Negative		39.94 (30.91)	72.01 (27.33)	84.27 (19.43)
		Men	Positive	69.32 (26.77)	61.27 (28.60)	63.40 (31.08)	79.46 (22.24)
			Negative		46.82 (30.26)	64.00 (29.72)	80.00 (22.44)
	Typical Use at Home	Women	Positive	69.11 (25.89)	65.05 (27.29)	69.33 (27.01)	79.85 (21.36)
			Negative		42.69 (31.33)	68.63 (26.37)	80.29 (21.60)
		Men	Positive	67.88 (26.98)	62.57 (28.00)	62.74 (29.21)	75.51 (23.70)
			Negative		49.13 (29.63)	62.65 (28.39)	75.63 (24.15)

Mean POMP scores are shown for each gender based on strategy, context, and emotional valence (when available). For the years in which questions did not specify emotional valence, only a single mean is shown. Standard deviations appear in parenthesis. The sample sizes for the studies are: Study 1—1,572 women and 1,900 men; Study 2—1,106 women and 1,353 men; Study 3—1,855 women and 2,135 men; and Study 4—2,987 women and 3,319 men.

#### Age

We had access to age in three of the four studies. We did not observe any significant interactions with age in Study 2 (all *p*s > 0.37). We observed an interaction between valence and age in Study 3, *F*_(1, 3988)_ = 10.18, *p* < 0.002, Cohen's *d* = 0.10; and Study 4, *F*_(1, 6304)_ = 25.01, *p* < 0.001, Cohen's *d* = 0.13. This was qualified by several three-way interactions that were quite small in effect size, but significant, and consistent across Studies 3 and 4. Specifically, we observed a significant interaction between self-reported regulation strategy, context, and age in Study 3, *F*_(1, 3988)_ = 4.30, *p* < 0.039, Cohen's *d* = 0.07; and Study 4, *F*_(1, 6304)_ = 38.84, *p* < 0.001, Cohen's *d* = 0.16. We also observed an interaction between self-reported regulation strategy, valence, and age as a trend in Study 3, *F*_(1, 3988)_ = 3.81, *p* = 0.05, Cohen's *d* = 0.06; and Study 4, *F*_(1, 6304)_ = 36.26, *p* < 0.001, Cohen's *d* = 0.15. Finally, we also observed an interaction between context, valence and age in Study 3, *F*_(1, 3988)_ = 4.78, *p* < 0.03, Cohen's *d* = 0.07; and Study 4 *F*_(1, 6304)_ = 4.59, *p* < 0.04, Cohen's *d* = 0.05. In all cases, the interactions we describe in the main analysis section above became weaker as age increases. More specifically, these interactions were primarily characterized by the greatest decreases in suppression at Burning Man for the youngest individuals (but no age differences for changes in reappraisal), relatively less suppression (but not reappraisal) of positive compared to negative emotion for the youngest individuals, and relatively less regulation of positive compared to negative emotion at Burning Man (compared to typically at home), for the youngest individuals.

For Study 4, these same three-way interactions were qualified by a four-way interaction between self-reported regulation strategy, context, valence, and age, *F*_(1, 6304)_ = 11.28, *p* = 0.001, Cohen's *d* = 0.08. This interaction was in the same direction as findings from Study 3: youngest individuals showed the lowest levels of suppression of positive emotion at Burning Man. More specifically, this interaction was characterized by a three-way interaction between context, valence and age for suppression, *F*_(1, 6304)_ = 9.58, *p* = 0.003, Cohen's *d* = 0.08, but there was no such significant interaction for reappraisal, *F*_(1, 6304)_ = 1.73, *p* = 0.189, Cohen's *d* = 0.03. For suppression, this three-way interaction was driven by an interaction between context and age for the suppression of positive emotion, *F*_(1, 6304)_ = 29.17, *p* < 0.001, Cohen's *d* = 0.14, but not negative emotion (*p* = 0.78). This interaction was characterized by greater decreases in self-reported suppression of positive emotion at Burning Man for younger, *t*_(3238)_ = 21.91, *p* < 0.001, Cohen's *d* = 0.74, compared to older, *t*_(3066)_ = 16.90, *p* < 0.001, Cohen's *d* = 0.61, participants. Means split by median age are listed in Table 3.

**Table 3:** **Impact of age on emotion regulation usage at Burning Man and at home**.

**Strategy**	**Context**	**Age**	**Valence**	**Study 2**	**Study 3**	**Study 4**
Suppression	Burning Man	Young	Positive	14.34 (22.13)	14.33 (23.13)	15.17 (24.05)
			Negative	43.97 (30.56)	49.90 (30.73)	51.75 (30.74)
		Old	Positive	14.42 (22.35)	17.74 (25.98)	18.21 (26.38)
			Negative	43.51 (30.93)	49.70 (31.48)	49.89 (30.59)
	Typical Use at Home	Young	Positive	19.39 (25.19)	21.92 (26.69)	22.06 (26.94)
			Negative	46.42 (30.55)	49.31 (29.19)	52.44 (28.88)
		Old	Positive	18.35 (24.58)	24.46 (28.48)	23.21 (27.94)
			Negative	46.07 (30.60)	48.71 (29.50)	50.67 (28.88)
Reappraisal	Burning Man	Young	Positive	63.59 (28.43)	66.71 (29.99)	82.10 (20.84)
			Negative		67.81 (28.63)	82.13 (21.54)
		Old	Positive	62.32 (28.44)	67.68 (30.30)	81.06 (20.74)
			Negative		67.63 (29.24)	81.90 (20.79)
	Typical Use at Home	Young	Positive	64.29 (27.75)	65.29 (28.09)	77.08 (23.42)
			Negative		64.95 (27.42)	77.14 (23.99)
		Old	Positive	63.09 (27.67)	66.42 (28.85)	78.08 (21.95)
			Negative		66.01 (27.87)	78.56 (22.10)

Mean POMP scores are shown for median-split age groups based on strategy, context, and emotional valence (when available). Age was not collected for Study 1. For the years in which questions did not specify emotional valence, only a single mean is shown. Standard deviations appear in parenthesis. The median age and sample sizes for each study are: Study 2—median age is 34.06 with 1,229 participants under that age and 1,229 participants older; Study 3—median age was 34, with 2,171 younger participants and 1,819 older participants; and Study 4—median age was 32 with 3,239 younger participants and 3,067 older participants. Statistical values reported in the text are based on age as a continuous variable; use of a median split for groups is done here for clearer presentation.

#### Previous experience with Burning Man

Because Burning Man is considered a relatively unique environment, we were interested in whether the relationships we previously reported are similar whether this was the participant's first year at the event, or if they had attended previously. In our samples, there were 1389 first-year participants for Study 1 (40%), 930 for Study 2 (37.8%), 1801 for Study 3 (45.1%), and 2709 for Study 4 (43%). We observed an interaction between self-reported strategy and previous experience with Burning Man in Study 2, *F*_(1, 2457)_ = 17.09, *p* < 0.001, Cohen's *d* = 0.17; Study 3, *F*_(1, 3988)_ = 5.27, *p* < 0.03, Cohen's *d* = 0.07; and Study 4, *F*_(1, 6304)_ = 15.05; *p* < 0.001, Cohen's *d* = 0.10. This interaction was characterized by lower levels of self-reported suppression in those with previous experience at Burning Man compared to those who were attending for the first time in Study 2, *t*_(2457)_ = 4.80, *p* < 0.001, Cohen's *d* = 0.19; Study 3, *t*_(3988)_ = 3.29, *p* < 0.002, Cohen's *d* = 0.10; and Study 4, *t*_(6304)_ = 3.87, *p* < 0.001, Cohen's *d* = 0.10. Indeed, those who had previously attended Burning Man reported suppressing less than first year attendees both at Burning Man in Study 2, *t*_(2457)_ = 5.17, *p* < 0.001, Cohen's *d* = 0.16; Study 3, *t*_(3988)_ = 3.98, *p* < 0.001, Cohen's *d* = 0.13; and Study 4 *t*_(6304)_ = 3.62, *p* < 0.001, Cohen's *d* = 0.09, and typically at home in Study 2, *t*_(2457)_ = 3.82, *p* < 0.001, Cohen's *d* = 0.15; Study 3, *t*_(3988)_ = 2.15, *p* < 0.05, Cohen's *d* = 0.07; and Study 4 *t*_(6304)_ = 3.63, *p* < 0.001, Cohen's *d* = 0.09. For self-reported reappraisal, we saw no differences between those with previous experience and first year attendees, all *p*s > 0.07.

In Study 3 alone, we observed a three-way interaction between self-reported strategy, context, and previous experience, *F*_(1, 3988)_ = 7.98, *p* < 0.006, Cohen's *d* = 0.09. This was characterized by an interaction between context and previous experience for suppression, *F*_(1, 3988)_ = 4.91, *p* < 0.03, Cohen's *d* = 0.07. The suppression interaction indicated that for Study 3, the differences reported above between previous and first-year attendees was slightly stronger at Burning Man than typically at home. There was only a trend for a context by previous experience interaction for reappraisal, *F*_(1, 3988)_ = 3.01, *p* = 0.08, Cohen's *d* = 0.05; and no comparisons between those with previous experience and first-year attendees were significantly different for reappraisal (all *p*s > 0.36). Also, previous experience did not interact with valence for self-reported suppression or reappraisal (all *p*'s > 0.23). Means split by previous experience are in Table 4.

**Table 4:** **Strategy use by previous Burning Man experience**.

**Strategy**	**Group**	**Study 1**	**Study 2**	**Study 3**	**Study 4**
Suppression	First-Year	43.62 (29.82)	33.24 (20.39)	35.61 (20.43)	36.55 (20.22)
	Previous Attendee	41.19 (29.48)	29.31 (19.29)	33.50 (19.88)	34.57 (20.21)
Reappraisal	First-Year	70.04 (24.73)	62.29 (26.86)	66.40 (23.77)	79.26 (19.76)
	Previous Attendee	68.68 (25.09)	63.95 (26.56)	66.63 (24.44)	80.12 (18.75)

Mean POMP scores for the two regulation strategies, reappraisal and suppression, are shown for first year Burning Man attendees and previous attendees. Standard deviations are shown in parentheses. The sample sizes for the studies are: Study 1—1,398 first-years and 2,083 previous attendees; Study 2—930 first-years, 1,529 previous attendees; Study 3—1,801 first-years, 2,189 previous attendees; and Study 4—2,709 first-years, 3,597 previous attendees.

## Discussion

To more fully describe changes in emotion regulation in different sociocultural contexts, we measured the self-reported use of expressive suppression and cognitive reappraisal in an alternative context (Burning Man; an annual week-long art festival) to see how sociocultural context influences the regulation of positive and negative emotion. This was an extension of a previous study that observed an interaction between emotion regulation strategy and sociocultural context (McRae et al., 2011). We replicated these findings and also observed a novel interaction between context, self-reported regulation strategy, and the valence of the emotion being regulated. In addition, we report differences in self-reported emotion regulation strategy use by gender, age, and previous experience with Burning Man.

### Changes in emotion regulation

Self-reported suppression of both positive and negative emotion decreased among participants who were in an alternative, temporary, rapidly changing, and openly expressive sociocultural context, filled with novel stimuli; however, decreases in the self-reported suppression of positive emotion were much stronger than those for negative emotion. Based on previous results alone (McRae et al., 2011), it was plausible that the adaptive decrease in suppression that occurs at Burning Man is primarily due to the decreased suppression of negative emotion, which would have relieved individuals from the paradoxical, maladaptive consequences of using suppression to attempt to decrease negative emotion (Gross, 1998a; Goldin et al., 2008). However, we observed more prominent decreases in the suppression of *positive* emotion at Burning Man. Individuals also reported suppressing negative emotion less often at Burning Man than typically at home, but the difference between the contexts is greater for positive emotion. This valence specificity increases our understanding of the precise ways that a sociocultural context can influence emotion regulation.

One of the benefits of decreased suppression usage in the Burning Man context is the allowance for increased positive emotion, which is likely to have individual, social, and cultural benefits. Individually, increased suppression of positive emotion is associated with decreased *experience* of positive emotion (Nezlek and Kuppens, 2008) so decreased suppression of positive emotion may lead to longer-lasting positive experiences. Socially, decreased suppression of positive emotion may facilitate the formation of new friendships and romantic relationships, as well as strengthen existing ones (Gross and John, 2003). Culturally, decreases in suppression of positive emotion may in turn facilitate the creation of a cultural environment that supports joyful experimentation among adults, like that which is enjoyed by children on playgrounds. The increased expression of positive emotion might encourage adults to play, by experimenting with new identities, emotional repertoires, senses of self, and cultural tools more than they would in other situations.

Previous research indicates that reappraisal has different properties when it is used to increase positive and decrease negative emotion (Shiota and Levenson, 2009, 2012; McRae et al., 2012), which would prove interesting if reappraisal were used more for one of these emotional goals than the other at Burning Man. However, we did not observe consistent differences in the change of self-reported reappraisal use between positive and negative emotion, even with a very large sample size. Therefore, people may enjoy the general benefits associated with using reappraisal more in this alternative sociocultural context, but we did not see changes that would suggest benefit increases related specifically to reappraising positive or negative emotions.

### Effects of a temporary sociocultural context

Only a couple of studies have looked at changes in emotion regulation by relatively local sociocultural context. One examined a standard transition between high school and college, a common transition between two typical social environments (Srivastava et al., 2009). The other study looked at Burning Man, a temporary social context and an alternative culture that thousands of people have visited, many of whom have an explicit countercultural intent to participate in the creation of a different kind of society, one that is less hierarchical and more joyful (McRae et al., 2011). The study on the transition to college found an *increased* use of suppression following the transition, whereas the previous Burning Man study found *decreases* in suppression and increases in reappraisal usage at Burning Man. Knowing that reduced suppression of positive emotion is the most prominent change in emotion regulation in this alternative context might help us better understand what aspects of this alternative sociocultural context contribute most strongly to changes in emotion regulation.

The social milieu at Burning Man is one environment that offers people an alternative model for emotion regulation. Everyday life in the US can be serious and subdued, infused with a Protestant work ethic promising that hard work will lead to salvation (Durkheim, 1933; Weber, 2008) and the need to express oneself in ways that work to maintain a positive image and story of oneself (Goffman, 1955; Wellington, 2001). Therefore, the consequences of transparent emotional expression may be unfavorable in everyday contexts in the US, including during the transition to college and in certain industries with expressive demands (Hochschild, 1983; Pierce, 1995; Srivastava et al., 2009). There are occasionally times and places for expressing oneself more freely, such as church revivals, spring break, Mardi Gras, Greek life parties, funerals, weddings, and other spiritual or religious mass gatherings. In these contexts, a person may feel that they can suspend normal emotional display rules and express emotions— perhaps even loudly. However, these opportunities can be short and/or infrequent, many occur in private spaces, and forms of expression are constrained by custom. In contrast, spontaneous, creative, and boisterous expressions of emotion are common in public spaces at Burning Man, even those that cause discord and interpersonal conflicts. A person may feel that they are able to express emotion more openly in this setting than typically, where it may be more likely that a person could be harshly judged for expressing emotion in ways that defy established norms. At Burning Man, where there are fewer rigid norms and new customs are still emerging, self-expression has become cherished as a public good, which encourages people to explore and experiment with many types of emotional expression. Unexpected, joyful outbursts are especially appreciated at Burning Man. This is not to say that everyone feels joyful at Burning Man all week long, in fact, grief and sorrow are openly expressed at the Temple, where participants inscribe messages on the walls about death, illness and potential trauma (Pike, 2005; Gilmore, 2010). But the data reported here demonstrate that if and when people feel positive emotion, they are more likely to express it publicly. The alternative environment provides a sociocultural context where expression is encouraged and reputational costs are lower, resulting in decreased suppression for all emotion, but especially for positive emotion.

As we have previously postulated, an alternative sociocultural context allows many participants to view their everyday lives from a broader, distanced perspective, which is a key ingredient in reappraisal (McRae et al., 2011). The novel social structure, including new social relationships, the gift economy, and focus on art may all encourage increased reappraisal of both positive and negative emotion. Along with previous findings, our findings show that sociocultural context can influence emotion regulation more quickly than originally thought. Though it is unclear what factors (e.g., radical self-expression, emphasis on artistic expression, etc.) at Burning Man account for these changes, it is possible that similar emotion regulation changes occur in other celebratory sociocultural contexts (e.g., Mardis Gras). It is still uncertain which types of contexts are more or less conducive to quick changes in emotion regulation. Through this particular social experiment, people seem to have discovered a way to increase their use of adaptive strategies for regulating both positive and negative emotion.

### Effects of gender, age, and previous experience at burning man

The consequences for expressing emotion may be unfavorable for everyday life in the US, more so for men than women, and more so for younger people than older individuals. Our examination of demographic variables indicated that contextual effects of self-reported suppression and reappraisal used to regulate positive and negative emotion are slightly different in different groups. Although these interactions were much smaller than those reported for the contextual effects, they hint at how this sociocultural context might influence individuals in different ways. Most prominently, the decreased suppression of positive emotion at Burning Man is strongest in men and younger adults. Previous work has demonstrated that men use suppression more than women in everyday life, and younger individuals use suppression more than older individuals in everyday life (John and Gross, 2004). Therefore, these groups might enjoy the greatest relative benefit of an alternative environment like Burning Man. In Studies 2–4, those who had attended Burning Man previously showed decreased suppression regardless of context. As the culture of Burning Man is spreading through regional events, Burning Man is indeed a social movement—and may indicate a resurgence of emotional expression in public. We observed the lasting impact of this movement when observed that individuals with previous experience at Burning Man reported decreased use of suppression—not only when at the event, but also at home in their typical lives. This means that although the event itself is temporary, the changes in self-reported emotion regulation we observed begin to take hold outside of the event as well. Future research should examine (1) if this is only true of those who choose to return to the event, or if these changes last even if someone has not visited Black Rock City for some time; and (2) the effects of experience at Burning Man on emotion regulation in a variety of other sociocultural contexts.

## Limitations and future directions

The present study replicated and extended previous findings with four separate samples of considerable size, but was not without limitations. Although Burning Man provides an excellent opportunity for the study of an alternative sociocultural context, the environment presented several challenges for data collection. First, because our items were on a longer survey, we were restricted by length, and could only add single self-report items to examine the use of each regulation strategy to influence each type of emotion, and strategy use for the typical home context was reported retrospectively. Though previous research indicates that single items can be reliable (Robins et al., 2001; Gosling et al., 2003), we plan to ask participants to make these ratings on multiple items, in each context in future years. Participants' reporting their use of these regulation strategies at home retrospectively presents two potential problems. The first is that the explicit culture of “radical self-expression” at Burning Man might contribute to demand effects, wherein participants report using suppression less often at Burning Man than at home. However, we feel that the distinction we observed between self-reported suppression of positive and negative emotion is still of interest. Additionally, the effects of reappraisal that we report are much less likely to be influenced by the demand of the Burning Man environment in the same way, as there are no explicit cultural values surrounding reappraisal. Second, because participants were estimating their strategy use typically at home retrospectively, these reports might be influenced by failures of memory or other biases. Until we measure self-reported regulation strategy use in both the Burning Man and typical home contexts, we are unable to rule out this potential source of error.

In addition, it is possible that the responses to the emotion regulation questions reported here were influenced by the presence of other questions that were asked on the questionnaire given each year. However, because the specific questions asked every year were not identical, we are confident that the effects we report are consistent across studies and reflect changes in self-reported emotion regulation as opposed to changes in the surrounding questions. Also, because our sample was one of volunteers (a convenience sample) we cannot ensure that they represent the population of Burning Man. In future years we plan on collecting a representative sample and using weighting techniques to adjust this data to be able to speak for the population as a whole. Finally, participants at Burning Man are exposed to a variety of challenges, including a hostile living environment, extreme weather, sleep deprivation, and dehydration, all of which have the potential to influence their state of mind and ability to fill out questionnaires accurately and conscientiously. In the present study, we excluded participants that did not demonstrate careful reading and responding to a quality control item, but it is possible that not all questions were answered thoughtfully.

For future studies it will be valuable to examine not only self-reported use of emotion regulation strategies but also direct reports of emotion experience. This will capture a broader picture, not of *attempts* to change positive and negative emotion, but the relative *success* of those efforts (for a discussion of emotion regulation frequency vs. success, see McRae, 2013). Previous studies have also shown that suppression and reappraisal can be used to both up- and down-regulate emotion. In future studies, it will be valuable to consider how successfully both positive and negative emotion can be up- and down- regulated by the strategies reported here.

## Conclusion

The present study extends previous research by showing that sociocultural context differentially influences how individuals regulate positive and negative emotion. According to participants' self report of emotion regulation at home and at Burning Man, an alternative sociocultural context that explicitly encourages “radical self-expression” is associated with decreased use of self-reported suppression of positive and negative emotion; but this is most strongly driven by the decreased suppression of positive emotion. By contrast, reappraisal increases comparably for both positive and negative emotion at Burning Man. These findings enhance our understanding of the effects of sociocultural context on the use of emotion regulation strategies that are known to be differentially adaptive. There are likely to be other contexts and occasions where cultural and social norms influence how individuals regulate emotion. These findings have implications for understanding the sociocultural contexts in which suppression, especially the suppression of positive emotion, and its associated maladaptive effects, may be minimized.

### Conflict of interest statement

The authors declare that the research was conducted in the absence of any commercial or financial relationships that could be construed as a potential conflict of interest.

## Footnotes

^1^A trend for a context by gender interaction was observed for Study 1, *F*_(1, 3470)_ = 3.12, *p* = 0.08, Cohen's *d* = 0.06; and Study 2, *F*_(1, 2457)_ = 3.16, *p* = 0.08, Cohen's *d* = 0.07. For both studies, this interaction was characterized by greater use of emotion regulation in men than women both at Burning Man in Study 1, *t*_(3470)_ = 5.90, *p* < 0.001, Cohen's *d* = 0.20; and Study 2, *t*_(2457)_ = 2.06, *p* < 0.001, Cohen's *d* = 0.08, and typically at home in Study 1, *t*_(3470)_ = 6.99, *p* < 0.001, Cohen's *d* = 0.24; and Study 2, *t*_(2457)_ = 3.28, *p* < 0.001, Cohen's *d* = 0.13, but the gender difference tends to be more pronounced typically at home.
